# Exploiting the RUSH System to Study Lytic Granule Biogenesis in Cytotoxic T Lymphocytes

**DOI:** 10.1007/978-1-0716-3135-5_27

**Published:** 2023-01-01

**Authors:** Nagaja Capitani, Chiara Cassioli, Keerthana Ravichandran, Cosima T. Baldari

**Keywords:** Intracellular trafficking, RUSH system, Cytotoxic granules, Granzyme B, Lytic granule biogenesis, Confocal microscopy, Spinning disk microscopy

## Abstract

The *Retention Using Selective Hooks* (RUSH) system allows for the synchronized release of one or more proteins of interest from a donor endomembrane compartment, usually the endoplasmic reticulum, and the subsequent monitoring of their traffic toward acceptor compartments. Here we describe the RUSH system applied to cytotoxic T cells to characterize the biogenesis of lytic granules, using as a proof-of-concept granzyme B trafficking to this specialized compartment.

## 1 Introduction

Cytotoxic T lymphocytes (CTLs) are the T cell effectors specialized for the elimination of cells infected by intracellular pathogens as well as cancerous cells. CTLs exert their cytotoxic function through two well-characterized cytotoxic mechanisms: release of cytotoxic granules (CG) and Fas/FasL mediated apoptosis [1]. The first mechanism involves the release of cytotoxic molecules from CGs via exocytosis at the immunological synapse (IS), a specialized contact area that forms at the interface of the T cell with its cognate target cell [2, 3]. Upon TCR-mediated recognition of a cell presenting specific peptide associated with MHCI, CTLs rapidly polarize and reorganize their cytoskeleton to translocate the microtubule-organizing center (MTOC) toward the synaptic interface [4, 5]. MTOC docking beneath the IS is a key step in the formation of a lytic synapse as it ensures the microtubule-assisted directional transport of CGs that contain soluble cytolytic proteins, such as granzymes and perforin, that are eventually released into the synaptic cleft [6, 7]. The other mechanism by which CTLs exert their killing activity is based on the FasL-dependent pathway. This mechanism involves cross-linking of the surface death receptor Fas on target cells induced by cell surface FasL expressed on CTLs. Fas/FasL interaction rapidly induces activation of the caspase cascade to promote target cell apoptosis [8].

A third mechanism of CTL-mediated killing has been recently reported by Balint and colleagues [9]. This is based on the synaptic release of a non-membranous type of cytotoxic particles, known as supramolecular attack particles (SMAPs) [9]. Proteomic and structural analysis of SMAPs revealed that together with canonical cytolytic components previously observed in CGs, such as granzyme B (GZMB) and perforin (Prf), this new class of particles is enriched in glycoproteins such as thrombospondin-1 (TSP-1) and galectin-1 (Gal1) that form a shell surrounding the cytotoxic core [9]. The recent in-depth characterization of CGs by Chang and colleagues has identified two populations of CGs, the “classical” single-core CGs (SCG) and the multicore CGs (MCG), which are the source of SMAPs [10].

The co-existence of SCGs and MCGs in CTLs and the fact that they are released independently supports the notion that their biogenesis and trafficking are governed by specific pathways. The development of a method to unravel these pathways will allow to achieve insight into the relationship between SCGs and MCGs and their interplay in CTL-mediated killing. In 2012, Boncompain and colleagues developed an assay known as Retention Using Selective Hooks (RUSH) to analyze the traffic of secretory proteins in eukaryotic cells [11].

This system is based on the simultaneous expression of two proteins: the hook used as “anchor” protein, and the reporter, the protein of interest whose trafficking will be analyzed. The hook is stably expressed in a donor compartment, usually the endoplasmic reticulum (ER), and the Golgi compartment. The hook is fused at its N- or C-terminal end with core streptavidin, which can be oriented toward the lumen of compartment or at the cytoplasmic face. The reporter is fused to the streptavidin-binding peptide (SBP) to allow its interaction with streptavidin on the hook protein and with a fluorescent protein (e.g., EGFP, mCherry) to visualize it within the cell.

**Fig. 1:**
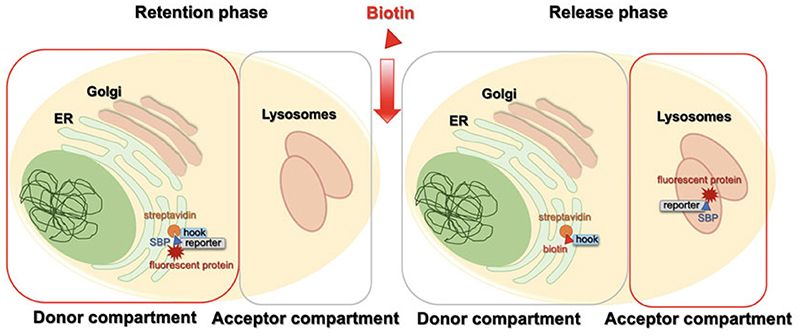
RUSH system. The RUSH (Retention Using Selective Hooks) system is a two-phase assay. In the initial phase, known also as “retention phase,” the reporter protein is anchored in the donor compartment (ER in the example) by the hook due to the streptavidin-SBP (streptavidin-binding peptide) interaction. Addition of biotin in the culture medium introduces the second phase of the assay, known as “release phase.” The reporter protein is released from the hook as the result of displacement by biotin and it traffics, together with the associated fluorescent protein, to its acceptor compartment (lysosomes in the example)

Once the RUSH construct is expressed by the cell, the reporter interacts with the hook in the donor compartment due to the streptavidin-SBP interaction and is thus retained in the donor compartment. When biotin is added to the culture medium, it efficiently binds streptavidin, displacing the SBP, and the reporter protein is released and can traffic toward its destination compartment, also known as acceptor compartment (Fig. 1).

This system can be applied to more than one reporter, allowing to track the synchronized trafficking of two proteins fused to different fluorescent tags. Here we describe the application of the RUSH system to the study of the biogenesis and intracellular trafficking of the CG components. As a proof-of-concept, we used the RUSH system to synchronize the traffic of granzyme B (GZMB), which is known to exploit the cation-independent mannose-6-phosphate pathway for specific targeting to CGs [12]. As a hook, we used the KDEL sequence, which retains the fused protein in the ER lumen [13]. To verify the correct localization to CGs, which are specialized lysosomes, we co-stained CTLs with the lysosomal marker LAMP-1 at the experiment endpoint. The results provide proof-of-concept that the RUSH system can be exploited to track the traffic of CG-associated proteins in CTLs. This paves the way to dissect the interplay of the pathways that regulate the biogenesis of SCGs and MCGs by synchronizing the trafficking of different CG components in CTLs co-transfected with RUSH constructs expressing the respective reporters.

## 2 Materials

### 2.1 Cloning of GZMB in a RUSH Construct

1. Commercial Addgene plasmid (Str-KDEL_TNF-SBP-mCherry, Plasmid #65279).
2. Phusion DNA Polymerase.
3. 5× Phusion HF Buffer (ThermoFisher scientific or equivalent).
4. dNTP Mix (10 mM each).
5. Primers containing restriction enzymes for amplification of the insert to be cloned in Addgene vector:
  Primer forward: TTGGCGCGCCATGCAACCAATCCTGCTTCTGCTGG
  Primer reverse: GAATTCCGGTAGCGTTTCATGGTTTTCTTTATCC
6. UltraPure DNase/RNase-Free Distilled Water.
7. Thermal cycler.
8. Restriction enzymes: FastDigest SgsI (AscI) and EcoRI (ThermoFisher scientific).
9. Agarose gel.
10. NucleoSpin Gel and PCR Clean-up Kit (Macherey-Nagel or similar).
11. UV-Vis spectrophotometer for μL volumes of nucleic acids (QIAxpert system (QIAGEN) and QIAxpert slides (QIAGEN) or similar).
12. Ligation: T4 DNA ligase 10× buffer and T4 DNA ligase.
13. *E*. *coli* thermocompetent cells (e.g., DH5α Competent Cells).
14. LB (Luria-Bertani) liquid medium (for each 950 mL of MilliQ H_2_O, add 10 g Tryptone, 10 g NaCl, and 5 g Yeast Extract) (*see* Note 1).
15. LB agar plates with 100 μg/mL ampicillin (see Note 2).
16. Petri dishes.
17. NucleoSpin Plasmid, Mini kit for plasmid DNA (Macherey-Nagel or similar).
18. NucleoBond Xtra Midi Plus EF, Midi kit for endotoxin-free plasmid DNA (Macherey-Nagel or similar).

### 2.2 CD8^+^ Cell Purification from Peripheral Blood and CTL Differentiation

1. RosetteSep Human CD8^+^ T cell Enrichment Cocktail (STEM-CELL Technologies or similar methods of purification of untouched CD8^+^ T cells).
2. Lympholyte Cell Separation Media or equivalent.
3. Phosphate buffered saline (PBS) (Medicago AB), 1 tablet in 1000 mL of deionized water (0.14 M NaCl, 0.0027 M KCl, 0.010 M Phosphate buffer, pH 7.4), sterile filtered.
4. Bovine Calf Serum (BCS)—0.1 μm sterile filtered and low endotoxin.
5. PBS with 0.2% (v/v) BCS: 2 mL of BCS and 98 mL of PBS (Medicago AB, see point 3), keep sterile.
6. High speed centrifuge, swing-bucket rotors, room temperature.
7. Transfer pipette.
8. R10 Medium: RPMI-1640 medium (Sigma-Aldrich) with 2 mM L-Glutamine and 20 mM HEPES, supplemented with 10% (v/v) BCS, 2 mg/mL penicillin, 1% (v/v) MEM Non-essential amino acids.
9. Recombinant human IL-2 IS, premium grade (Miltenyi Biotec) (*see* Note 3).
10. Dynabeads Human T-activator CD3/CD28 for T cell Expansion and Activation (ThermoFisher Scientific or equivalent) (see Note 4).
11. Magnetic particle concentrator MCP-6 (Dynal or equivalent).
12. PBS 0.1% (v/v) BCS: 1 mL of BCS and 99 mL of PBS, keep sterile.
13. 12-well cell culture plate with flat bottom.

### 2.3 CTLs Nucleofection with the RUSH-GZMB Construct

1. Amaxa T cell nucleofector kit (Lonza).
2. Nucleofector 2b Device (Lonza).

### 2.4 Evaluation of RUSH on Fixed Cells by Confocal Microscopy

1. CTLs transfected with RUSH-GZMB construct.
2. Cell culture plate, 96 well.
3. 4 mM biotin (stock solution) (*see* Note 5).
4. 10-well diagnostic microscope slides.
5. Absolute ethanol.
6. 0.1% (w/v) Poly-L-Lysine (PLL) stock solution in sterile water (Sigma-Aldrich or similar).
7. Hellendahl-type dish.
8. 3MM paper.
9. Fixation solution: 4% (v/v) paraformaldehyde (PFA) in PBS (*see* Note 6).
10. Permeabilization solution: PBS with 1% (w/v) BSA and 0.01% (v/v) Triton X-100].
11. Primary antibodies: anti-RFP, rabbit polyclonal and anti-RFP mouse monoclonal (Rockland); anti-human CD107a (LAMP-1), mouse monoclonal (BioLegend), anti-CanX, rabbit polyclonal (Sigma-Aldrich).
12. Secondary fluorochrome-conjugated antibodies.
13. Slide mounting medium: 90% (v/v) glycerol in PBS. Coverslips 24 × 60 mm and conventional nail polish.
14. Confocal microscopy, Zeiss LSM700.

### 2.5 Evaluation of RUSH in Live Cells

1. CTLs transfected with RUSH-GZMB construct.
2. LysoTracker Blue DND-22 (ThermoFisher Scientific).
3. 4 mM biotin (stock solution).
4. μ-Slide 8 Well Chamber Slide.
5. Medium RPMI 1640 no phenol red.
6. Spinning disk microscope with an inverted objective and a thermostated incubation chamber (CSU-W1-SoRa, Nikon, or equivalent).

## 3 Methods

### 3.1 Generation of the RUSH-GZMB Construct

PCR amplification of *GZMB* is performed using as template a GZMB-mCherry home-made vector.

PCR reaction mix (final volume 100 μL) (*see* Note 7):

#### 3.1.1 GZMB Amplification by PCR

20 μL 5× Phusion HF Buffer

2 μL 10 mM dNTPs

0.5 μM (final concentration) forward primer

0.5 μM (final concentration) reverse primer

50 ng template DNA

1 μL Phusion High–Fidelity DNA Polymerase

Add H_2_0 to 100 μL

PCR protocol:

Initial Denaturation 98 °C, 30 sec

$\begin{array}{l} Denaturation98{}^{\circ}C,10sec\\ Annealing68{}^{\circ}C,30sec\}35cycles\\ Extension72{}^{\circ}C15–30s/kb \end{array}$

Final extension 72 °C, 7 min

Hold 4°C

5 μL of the PCR product is run on an agarose gel (*see* Note 8) to verify the quality of PCR amplification. The remaining part is purified using NucleoSpin Gel and PCR Clean-up Kit and quantified using QIAxpert system (*see* Note 9).

#### 3.1.2 Insert and Vector Restriction Enzyme Digestion

Purified *GZMB* PCR product obtained in paragraph 3.1.1 and Addgene Plasmid #65279 are digested using the following protocol (*see* Note 10):

6 μL 10× FastDigest Buffer

2 μL FastDigest SgsI

2 μL FastDigest EcoRI template DNA (0.4 μg purified PCR product or 2 μg vector) Add H_2_0 to 60 μL

Digestion protocol: 30 min at 37 °C and 5 min at 80 °C

The digested products are loaded onto agarose gels, the DNA fragments are excised from the gel and purified using NucleoSpin Gel and PCR Clean-up Kit and quantified using QIAxpert system.

#### 3.1.3 DNA Ligation and Transformation

Digested inserts and vectors obtained in Subheading 3.1.2 are ligated using the following proportion

$$\frac{ngofvector\times kbsizeofinsert}{kbsizeofplasmid}\times molarratio\frac{insert}{plasmid}=nginsert$$

where the amount of vector for a ligation reaction is usually 50–100 ng and the molar ratio vector:insert varies depending on vector and insert size. In our ligation reactions we used 100 ng vector and a molar ratio of 3 (insert): 1 (vector) to obtain Str-KDEL_GZMB-SBP-mCherry (*see* Note 11).

Assemble the following reaction in a sterile microcentrifuge tube (*see* Note 12):

100 ng vector DNA

50–100 ng insert DNA

1 μL 10× Buffer

1 μL T4 DNA Ligase

Nuclease-free water to 10 μL

Incubate the reaction at room temperature for 3 h, or 4 °C overnight, and inactivate the reaction by heating at 70 °C for 10 min.

The ligation reaction obtained is transformed in *E*. *coli* DH5α Competent Cells following this procedure:

1. Thaw on ice one tube of DH5α cells.
2. Gently mix cells with the pipette tip and aliquot 50 μL cells for each transformation reaction in a 1.5 mL microcentrifuge tube.
3. Add the ligated DNA (10 μL) to the cells and mix gently.
4. Incubate tubes on ice for 30 min.
5. Heat shock cells for 30 s in a 42 °C water bath.
6. Place back tubes on ice for 2 min.
7. Add 850 μL pre-warmed LB medium.
8. Incubate tubes in shaking incubator at 37 °C for 1 h at 225 rpm.
9. Spread 20 μL to 200 μL from each transformation on pre-warmed selective plates (*see* Notes 13 and 14).
10. Incubate plates overnight at 37 °C.

The next day pick single colonies and inoculate into LB medium. Incubate at 37 °C in shaking incubator at 225 rpm for 18–20 h.

#### 3.1.4 Plasmid DNA Preparation and Colony Screening

Bacterial cells obtained from single colonies are used to extract the plasmid using the NucleoSpin Plasmid Mini kit for plasmid DNA (Macherey-Nagel or similar). Plasmid DNA obtained (~200–300 ng) is digested with restriction enzymes as described in Subheading 3.1.2 and loaded onto agarose gels to verify the quality of ligated DNA. Plasmid DNA containing the correct insert is selected and a Midiprep is prepared from it using the Nucleo-Bond Xtra Midi Plus EF, Midi kit for endotoxin-free plasmid DNA (Macherey-Nagel or similar).

### 3.2 CD8^+^ Cell Purification from Peripheral Blood and CTL Differentiation

**Fig. 2:**
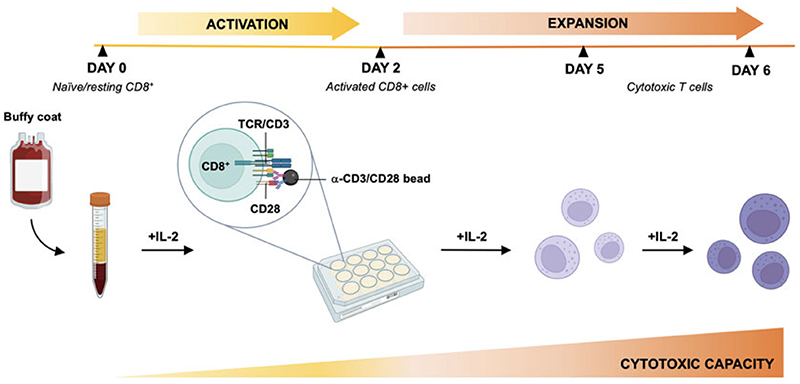
Schematic representation of a method to obtain differentiated CTLs starting from human naïve/resting CD8^+^ cells purified from buffy coats of healthy donors. Freshly isolated naïve/resting CD8^+^ cells are activated with commercial Dynabeads (without the need of antigen or antigen presenting cells) and expanded for 5–7 days to generate CTLs acquiring cytotoxic activity. CTLs were transfected 6 days after activation and analyzed at day 7. (The figure was partially generated with BioRender.com)

1. Isolate peripheral blood CD8^+^ T cells from buffy coats of healthy donors by negative selection (>95%) using the RosetteSep Human CD8^+^ T cell Enrichment Cocktail and subsequent centrifugation over Lympholyte Cell Separation Media. Briefly, add 50 μL of RosetteSep Human CD8^+^ T cell Enrichment Cocktail per mL of whole blood sample, mix, and incubate at room temperature for 20 min. Dilute the sample with PBS 2% (v/v) BCS and layer the diluted sample on a density gradient medium, being careful to minimize their mixing.
2. Centrifuge without brake. Carefully harvest the enriched cell layer with a transfer pipette and transfer to a new centrifuge tube. Wash enriched cells 2× with PBS 2% (v/v) BCS and proceed with the differentiation protocol.
3. Activate and expand resting CD8^+^ T cells (day 0) at a cell density of 0.5–2.5 × 10^6^ cells/mL in complete R10 medium with 50 U/mL rIL-2 (*see* Note 15).
4. Resuspend the Dynabeads Human T-activator CD3/CD28 in the vial, transfer the desired volume (12.5 μL of beads per 1 × 10^6^ of CD8^+^ T cells) to a tube, add at least 1 mL of PBS 0.1% (v/v) BCS, and mix. Place the tube on a magnet for 1 min and discard the supernatant. Remove the tube from the magnet and resuspend the washed Dynabeads in the same volume of complete R10 medium as the initial volume of Dynabeads taken from the vial.
5. Add washed Dynabeads to CD8^+^ T cells (cell-to-bead ratio = 1: 0.5).
6. Seed the cell/bead suspension at a cell density of 1 × 10^6^ cells/mL into a flat-bottom 12-well cell culture plate (2 mL/well) and incubate in a humidified CO_2_ incubator at 37 °C (see Note 16). 48 h after activation remove the beads (day 2) (see Note 17) and expand the cells in complete R10 medium with 50 U/mL rIL-2 for further 3 days (day 5).
7. Split the cells back to a density of 1×10^6^ cells/mL in complete R10 medium with 30 U/mL rIL-2 the day before nucleofection (Fig. 2).

### 3.3 CTL Nucleofection with the RUSH-GzmB Construct

At day 6, count the CTLs obtained and determine cell density. CTL nucleofection with the RUSH-GZMB construct is performed using the Amaxa T cell nucleofector kit, with the following protocol:

1. Centrifuge 2×10^6^ cells per sample at 200×g for 10 min at room temperature.
2. Discard supernatant completely and resuspend the cell pellet carefully in 100 μL room temperature Human T Cell Nucleofector Solution (*see* Note 18).
3. Combine 100 μL of cell suspension with 1.5 μg DNA (Str-KDEL_GZMB-SBP-mCherry).
4. Transfer cell/DNA suspension Amaxa certified cuvette (*see* Note 19).
5. Insert the cuvette with the cell/DNA suspension into the Nucleofector Cuvette Holder and apply the T-023 Nucleofector Program.
6. Add ~500 μL of the pre-equilibrated culture media to the cuvette and gently transfer the sample into a 12-well plate (final volume 2 mL media/well/sample) (*see* Note 20).

### 3.4 Evaluation of RUSH on Fixed Cells by Confocal Microscopy

**Fig. 3:**
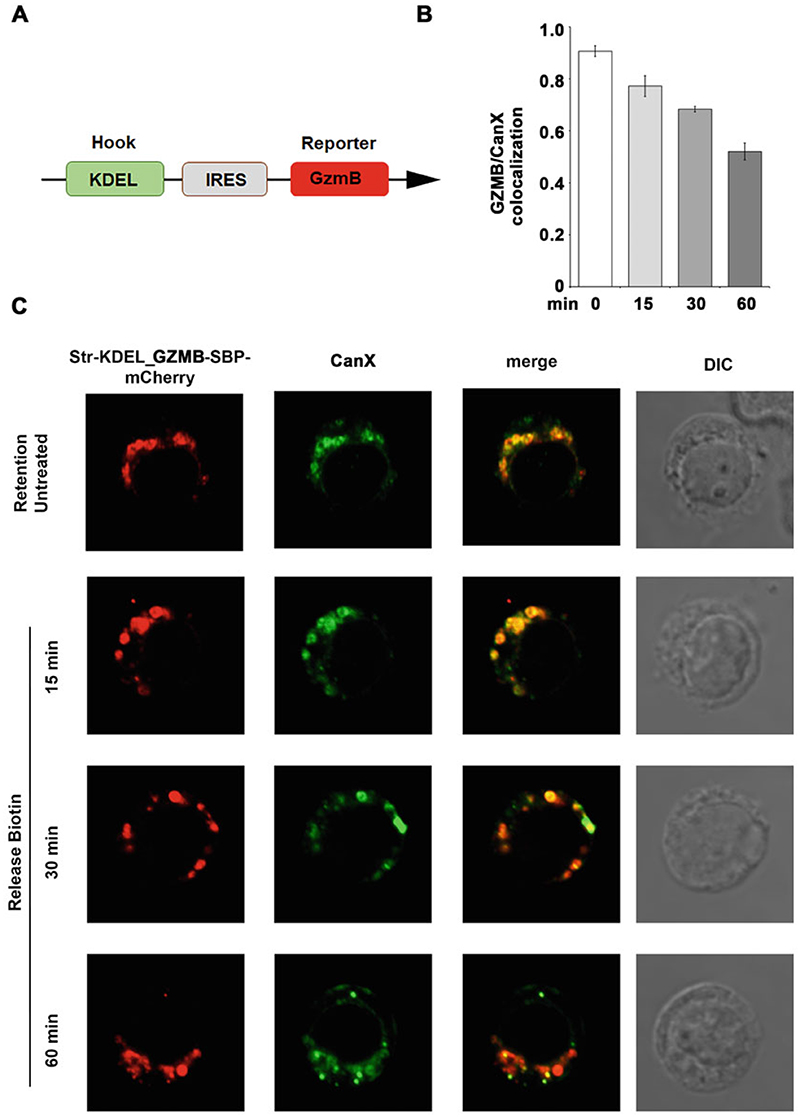
Reporter release from the “donor” compartment. (**a**) Schematic representation of a RUSH plasmid with the hook placed upstream of the IRES (internal ribosome entry site) and the reporter is downstream. (**b, c**) Human primary CTLs expressing Str-KDEL_GZMB-SBP-mCherry (red) and stained for the ER marker CanX Ab (green), with and without biotin treatment. Scale bar, 5 μm (c) Co-localization analyses of Str-KDEL_GZMB-SBP-mCherry with CanX were obtained using Manders’ coefficient (mean ± SD; 10 cells/sample) (**b**). The results show that before biotin addition GZMB strongly co-localizes with the ER, while after biotin addition the co-localization decreases in a time-dependent fashion, indicating release from the ER

**Fig. 4:**
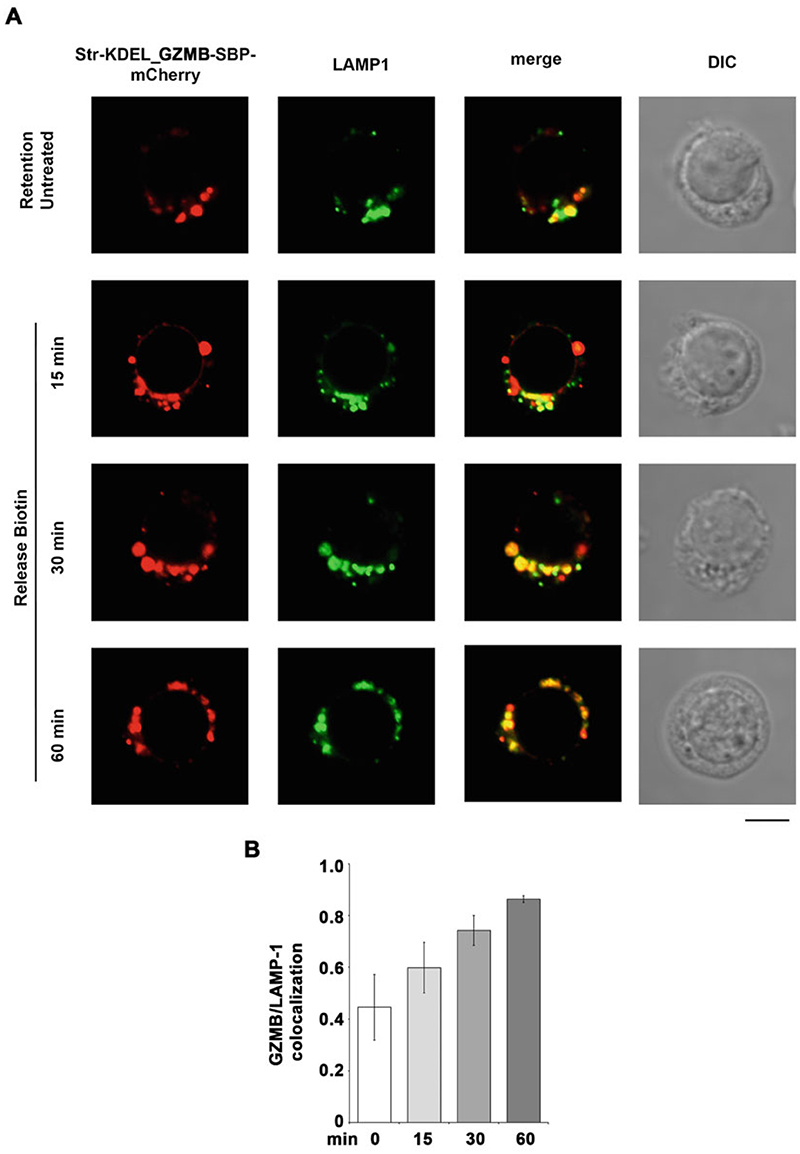
Reporter trafficking toward the “acceptor” compartment. (**a**) Human primary CTLs expressing Str-KDEL_GZMB-SBP-mCherry (red) and stained with anti-LAMP-1 Ab (green) with and without biotin treatment. Scale bar, 5 μm. (**b**) Co-localization analyses of Str-KDEL_GZMB-SBP-mCherry with LAMP-1 were obtained using Manders’ coefficient (mean ± SD; 10 cells/sample). The results show that before biotin addition GZMB has a low co-localization with lytic granules (marked by LAMP-1), while after biotin addition the co-localization increases in a time-dependent fashion, indicating that after release from the ER GZMB is correctly transported to its destination compartment

1. Cell preparation: collect the nucleofected CTLs and seed 10^5^ cells in a 96-well cell culture plate in a volume of 200 μL R10 medium/sample and incubate at 37 °C, 5% CO_2_.
2. Perform a time-course of release with biotin: plan a time-course for the reporter release, depending on the reporter that you are analyzing. In this protocol, we apply the following time-course: 0–15 min–30 min–60 min where the sample “0” is without biotin addition while the other time points indicate the time of biotin treatment. Add 40 μM biotin (from biotin 4 mM stock solution) to the treated samples. At the end of the time course, wash the samples with PBS and resuspend each sample in 30 μL PBS.
3. In the meantime, wash 10-well diagnostic microscope slides with absolute ethanol and allow them to dry. Coat each well with 300 μL of 0.01% PLL (1:10 dilution of stock solution in sterile water; *see* Note 21) and keep at RT for 20 min in the dark, then discard the solution and carefully wash with MilliQ. Air-dry the slides and keep in the dark.
4. Transfer all samples to the PLL-coated slides, one sample/well, and allow samples to adhere for 15 min at RT.
5. Remove the supernatant using a pipette (*see* Note 22), add 4% (v/v) PFA and keep for 20 min at RT in the dark.
6. Wash slides by immersion into a Hellendahl-type dish containing PBS. Dry the spaces between wells using 3 MM paper, being careful to not completely remove PBS from the wells to avoid cell disruption.
7. Add permeabilization solution [PBS with 0.1% (w/v) BSA and 0.01% (v/v) Triton X-100] dropwise to the wells and incubate for 20 min at RT.
8. Wash slides (as described in **step 5**).
9. Add 15 μL/well of anti-CanX antibody diluted 1:50 or anti-LAMP1 antibody diluted 1:400 mixed with anti-RFP antibody (*see* Note 23) diluted 1:500 in PBS and keep the slide in a humidified chamber at 4 °C overnight.
10. The next day wash slides (as described in **step 5**).
11. Add 15 μL/well of a mix of AlexaFluor secondary antibodies, diluted 1:80 each in PBS and keep the slide in a humidified chamber at RT for 45 min (*see* Note 24).
12. Wash slides (as described in **step 5**).
13. Add 90% (v/v) glycerol in PBS, one drop/well (*see* Note 25), and then overlay the coverslip, remove the excess of slide mounting solution by very carefully pushing onto the coverslip, then fix it using conventional nail polish.
14. Store the slides at 4 °C in the dark.
15. Analyze fluorescence on a confocal microscope to determine the localization of the protein of interest fused to the fluorescent tag. In our setting, where the hook is specific for the ER, the protein of interest should be localized in the ER in the absence of biotin (Fig. 3) and reach its destination compartment when biotin is added to the medium, depending on the kinetics of trafficking of the protein itself (Fig. 4). An example of the results for CTLs transfected with the Str-KDEL_GZMB-SBP-mCherry RUSH constructs is shown in Figs. 3 and 4.

### 3.5 Evaluation of RUSH on Live Cells

1. Preparation of μ-Slide 8 Well Chamber Slide: coat each required well with 200 μL of PLL and maintain at RT for 20 min in the dark, then discard the solution and carefully wash with MilliQ. Air-dry the slide and lodge it in the thermostated incubation chamber of the microscope.
2. Cell preparation: collect the nucleofected CTLs and seed 2×10^5^ cells in 200 μL of Medium RPMI 1640 no phenol red per well in PLL-coated μ-Slide 8 Well Chamber Slide.
3. Add immersion oil to the 60× objective and wait for transfected cells to adhere to the PLL-coated well.
4. Choose the appropriate configuration of your microscope for the fluorescent protein to be imaged. Adjust the focus and mark positions of interest.
5. Add 40 μM biotin (from biotin 4 mM stock solution) to the sample.
6. Acquire images with a time interval of 5 min for a total time of 60 min at 37 °C.
7. During long-term acquisition, having the following precautions: use the Perfect Focus System and prevent photobleaching by using low laser intensity and short exposure time whenever possible.

## 4 Notes

1. LB liquid medium composition is described in paragraph 2.1. Combine the reagents and shake until the solutes have dissolved. Adjust the pH to 7.0 and the final volume of the solution to 1 L with H_2_O. Sterilize by autoclaving.
2. LB agar plates with antibiotic selection are obtained as described for the LB liquid medium with the 15 g bacteriological agar per 1 L of water. Mix well by inverting the bottle several times until powder is dissolved and sterilize by autoclaving. Following autoclaving (while media is still liquid but cool enough to safely handle the bottle), add the desired antibiotic at the final concentration of 100 μg/mL and pour LB agar into Petri dishes. When agar has set, replace lid, invert plates, and store at 4 °C.
3. Resuspend lyophilized human IL-2 IS with deionized sterile-filtered water to a final concentration of 500 U/μL (corresponding to 0.1 mg/mL), and store aliquots at –20 °C or below. Avoid repeated freeze-thaw cycles.
4. Resuspend the Dynabeads carefully before use by vortexing the vial for >30 s.
5. Prepare a 4 mM biotin stock solution by dissolving 48 mg biotin in 50 mL culture medium.
  Filter using a 0.22 μm membrane to sterilize the solution and store up to 3 months at 4 °C.
6. 4% (v/v) PFA solution is prepared diluting a stock solution (16% PFA) in PBS1×, in sterile conditions, in the dark. Freshly 4% (v/v) PFA is prepared for each experiment.
7. Thaw on ice all the PCR reagents. Prepare a 10 μM primer solution in sterile water from a 100 μM stock. Be careful drawing the DNA Polymerase, because of its viscosity.
8. For gels, agarose is commonly used at concentrations of 0.7–2% depending on the size of bands to be separated. For example, to prepare a 1% (w/v) agarose gel 0.5 g of agarose in 50 mL 1× TAE (50× TAE buffer: 242.28 g Tris, 18.61 g EDTA Disodium, 57.1 mL Glacial Acetic Acid, dH2O up to 1 L). Mix agarose powder with 100 mL 1×TAE in a beaker. Microwave for 1–3 min until the agarose is completely dissolved, then let agarose solution cool down to about 50 °C and add 5 μL GelRed Nucleic Acid Gel Stain.
9. Pipet 2 μL of each sample for up to 16 samples onto a QIAx-pert Slide avoiding bubbles.
10. Thaw on ice the FastDigest Buffer and keep the enzymes on ice throughout the experiment. Be careful drawing the enzymes, because of their viscosity.
11. Insert:vector ratio is calculated on the base of insert and vector length (kb) and mass. The ideal ratio is usually 3:1, because a larger amount of insert increases the chances of successful cloning reaction.
12. Thaw on ice all the ligation reagents. Be careful drawing the T4 DNA ligase, because of its viscosity.
13. Plates should be removed from 4 °C at least 1 h before use and placed in 30 °C incubator.
14. Plating two different volumes of bacterial culture is recommended to ensure that at least one plate will have well-spaced colonies.
15. Split the cells when cell density exceeds 2.5 × 10^6^ cells/mL or when the medium turns yellow.
16. Examine cell culture daily, noting changes in cell size and shape.
17. Upon activation, some cells will bind strongly to the beads. Resuspend and transfer the bead/cell suspension to a suitable tube by thoroughly pipetting before cell separation from the beads on a magnet.
18. Avoid storing the cell suspension longer than 20 min, as this reduces cell viability and gene transfer efficiency.
19. Sample must cover the bottom of the cuvette without air bubbles.
20. Use the supplied pipettes and avoid repeated aspiration of the sample.
21. 0.1% (w/v) PLL stock solution is diluted 1:10 in distilled water and used fresh.
22. Do not completely dry the wells to avoid cell disruption.
23. The use of anti-RFP antibody in immunofluorescence experiments performed on cells transfected with Str-KDEL_TNF-SBP-mCherry construct permits to amplify the mCherry signal, if required.
24. The fluorescently labelled secondary Ab must be kept safe from the light. For this reason, prepare dilutions and perform incubations in the dark.
25. Dispense slide mounting medium dropwise onto the slide wells using a Pasteur pipette.

## References

[1] Cassioli C, Baldari CT (2022). The expanding arsenal of cytotoxic T cells. Front Immunol.

[2] Griffiths GM, Tsun A, Stinchcombe JC (2010). The immunological synapse: a focal point for endocytosis and exocytosis. J Cell Biol.

[3] Kabanova A, Zurli V, Baldari CT (2018). Signals controlling lytic granule polarization at the cytotoxic immune synapse. Front Immunol.

[4] Huse M (2012). Microtubule-organizing center polarity and the immunological synapse: protein kinase C and beyond. Front Immunol.

[5] Ritter AT, Asano Y, Stinchcombe JC, Dieckmann NMG, Chen B, Gawden-Bone C (2015). Actin depletion initiates events leading to granule secretion at the immunological synapse. Immunity.

[6] Stinchcombe JC, Majorovits E, Bossi G, Fuller S, Griffiths GM (2006). Centrosome polarization delivers secretory granules to the immunological synapse. Nature.

[7] Stinchcombe JC, Griffiths GM (2007). Secretory mechanisms in cell-mediated cytotoxicity. Annu Rev Cell Dev Biol.

[8] Bossi G, Griffiths GM (1999). Degranulation plays an essential part in regulating cell surface expression of Fas ligand in T cells and natural killer cells. Nat Med.

[9] Balint S, Müller S, Fischer R, Kessler BM, Harkiolaki M, Valitutti S (2020). Supra-molecular attack particles are autonomous killing entities released from cytotoxic T cells. Science.

[10] Chang H, Schirra C, Ninov M, Hahn U, Ravichandran K, Krause E (2022). Identification of distinct cytotoxic granules as the origin of supramolecular attack particles in T lymphocytes. Nat Commun.

[11] Boncompain G, Perez F (2012). Synchronizing protein transport in the secretory pathway. Curr Protoc Cell Biol.

[12] Griffiths GM, Isaaz S (1993). Granzymes a and B are targeted to the lytic granules of lymphocytes by the mannose-6-phosphate receptor. J Cell Biol.

[13] Boncompain G, Divoux S, Gareil N, de Forges H, Lescure A, Latreche L (2012). Synchronization of secretory protein traffic in populations of cells. Nat Methods.

